# Angioscopic Evaluation of Stabilizing Effects of Bezafibrate on Coronary Plaques in Patients With Coronary Artery Disease

**DOI:** 10.1155/DTE.7.21

**Published:** 2000

**Authors:** Yasumi Uchida, Yoshiharu Fujimori, Hidefumi Ohsawa, Jyunichi Hirose, Hirofumi Noike, Keiichi Tokuhiro, Masahito Kanai, Masaki Yoshinuma, Kazuhito Mineoka, Takashi Hitsumoto, Kaneyuki Aoyagi, Takeshi Sakurai, Shin Sato, Kokushi Yoshinaga, Masaaki Ozegawa, Hiroshi Morio, Katsumi Yamada, Kimiko Terasawa, Yuuko Uchida, Tomomitsu Oshima

**Affiliations:** 1 Clinical Physiology and Cardiovascular Center Toho University Hospital at Sakura Shimoshizu Sakura Chiba-Ken Japan; 2 Cardiology Narita Red Cross Hospital Iida Narita Chiba-Ken Japan; 3 Cardiology Funabashi-Futawa Hospital Futawa Higashi Funabashi Chiba-Ken Japan

## Abstract

*Background* Since long-term administrations of anti-hyperlipidemic agents result in reduction in  % stenosis or increase in minimum lumen diameter (MLD) of stenotic coronary segments, it is generally believed that anti-hyperlipidemic agents stabilize vulnerable coronary plaques. However, recent pathologic and angioscopic studies revealed that vulnerability of coronary plaques is not related to severity of stenosis and the rims rather than top of the plaques disrupt, and therefore, angiography is not adequate for evaluation of vulnerability.

*Angioscopy* enables macroscopic pathological evaluation of the coronary plaques. Therefore, we carried out a prospective angioscopic open trial for evaluation of the stabilizing effects of bezafibrate on coronary plaques.

*Methods* From April, 1997 to December, 1998, 24 patients underwent coronary angioscopy of the plaques in the non-targeted vessels during coronary interventions and 6 months later. The patients were divided into control (10 patients, 14 plaques) and bezafibrat (14 patients, 21 plaques) groups. Oral administration of bezafibrate (Bezatol SR, 400mg/day) was started immediately after the interventions and was continued for 6 months. The vulnerability score was determined based on angioscopic characteristics of plaques and it was compared before and 6 months later.

*Results*  Six months later, vulnerability score was reduced (from 1.6 to 0.8;*p* < 0.05) in bezafibrate group and unchanged (from 1.4 to 1.3; NS) in control group. In bezafibrate group, the changes in vulnerability score was not correlated with those in % stenosis or MLD.

*Conclusion* The results indicate that bezafibrate can stabilize coronary plaques.


*Diagnostic and Therapeutic Endoscopy*, Vol. 7, pp. 21–27
Reprints available directly from the publisher
Photocopying permitted by license only

© 2000 OPA (Overseas Publishers Association) N.V.
Published by license under
the Harwood Academic Publishers imprint,
part of The Gordon and Breach Publishing Group.
Printed in Malaysia.

### Angioscopic Evaluation of Stabilizing Effects of Bezafibrate on Coronary Plaques in Patients with Coronary Artery Disease

YASUMI UCHIDA[a,*], YOSHIHARU FUJIMORI[b], HIDEFUMI OHSAWA[a], JYUNICHI HIROSE[c], HIROFUMI NOIKE[a], KEIICHI TOKUHIRO[a], MASAHITO KANAI[a], MASAKI YOSHINUMA[a], KAZUHITO MINEOKA[a], TAKASHI HITSUMOTO[a], KANEYUKI AOYAGI[a], TAKESHI SAKURAI[a], SHIN SATO[a], KOKUSHI YOSHINAGA[a], MASAAKI OZEGAWA[b], HIROSHI MORIO[b], KATSUMI YAMADA[b], KIMIKO TERASAWA[b], YUUKO UCHIDA[b] and TOMOMITSU OSHIMA[c]

[a]*Clinical Physiology and Cardiovascular Center, Toho University Hospital at Sakura, Shimoshizu, Sakura, Chiba-Ken, Japan;* [b]*Cardiology, Narita Red Cross Hospital, Iida, Narita, Chiba-Ken, Japan;* [c]*Cardiology, Funabashi-Futawa Hospital, Futawa Higashi, Funabashi, Chiba-Ken, Japan*

*(Received 24 March 2000; Revised 29 May 2000; In final form 15 June 2000)*

***Background*** **Since long-term administrations of anti-hyperlipidemic agents result in reduction in % stenosis or increase in minimum lumen diameter (MLD) of stenotic coronary segments, it is generally believed that anti-hyperlipidemic agents stabilize vulnerable coronary plaques. However, recent pathologic and angioscopic studies revealed that vulnerability of coronary plaques is not related to severity of stenosis and the rims rather than top of the plaques disrupt, and therefore, angiography is not adequate for evaluation of vulnerability.**

***Angioscopy*** **enables macroscopic pathological evaluation of the coronary plaques. Therefore, we carried out a prospective angioscopic open trial for evaluation of the stabilizing effects of bezafibrate on coronary plaques.**

***Methods*** **From April, 1997 to December, 1998, 24 patients underwent coronary angioscopy of the plaques in the non-targeted vessels during coronary interventions and 6 months later. The patients were divided into control (10 patients, 14 plaques) and bezafibrate (14 patients, 21 plaques) groups. Oral administration of bezafibrate (Bezatol SR, 400 mg/day) was started immediately after the interventions and was continued for 6 months. The vulnerability score was determined based on angioscopic characteristics of plaques and it was compared before and 6 months later.**

***Results*** **Six months later, vulnerability score was reduced (from 1.6 to 0.8; $p < 0.05$) in bezafibrate group and unchanged (from 1.4 to 1.3; NS) in control group. In bezafibrate group, the changes in vulnerability score was not correlated with those in % stenosis or MLD.**

***Conclusion*** **The results indicate that bezafibrate can stabilize coronary plaques.**

*Keywords*: Angioscopy, Bazafibrate, Coronary plaque, Stabilization

* Corresponding author. Tel.: +81-43-462-8811. Fax: +81-43-487-0481.

### INTRODUCTION

Since long-term administrations of anti-hyperlipidemic agents result in reduction of cardiac events and increases survival rate, it is generally believed that anti-hyperlipidemic agents stabilize vulneable coronary plaques, however without definite evidence [1–3]. Coronary angiographic follow-up studies also demonstrated that their beneficial effects are not necessarily accompanied by significant reduction in severity of coronary stenoses [4–7].

Recent pathologic and angioscopic studies revealed that vulneability of coronary plaques is not related to severity of stenoses and the rims rather than top of the plaques disrupt [8,9]. Also, angioscopic studies in acute coronary syndromes demonstrated that plaques in less stenotic portions disrupt in a certain group of patients [10]. Therefore, angiography is not adequate for evaluation of vulnerability.

Angioscopy enables macroscopic pathological evaluation of the coronary plaques. Therefore, we carried out a prospective angioscopic open trial for evaluation of the stabilizing effects of bezafibrate on coronary plaques in patients who underwent coronary angioplasty.

### SUBJECTS AND METHODS

### Angioscopes and their Manipulation

Angioscopes used in this study were 4.7 F in external diameter with an inflatable balloon at the most tip (Clinical Supply Co, Gifu, Japan). A fiberscope (image guide 3000 or 6000 pixels and light guide 200 pixels) was included in a guiding catheter. For observation, balloon was inflated with carbon dioxide and body temperature heparinized (10 U/ml) saline was infused manually or when necessary by a power injector at a rate of 2–3 ml/sec. The fiberscope can be advanced over a 0.014 inch guide wire for up to 5 cm while the guiding catheter was fixed. Therefore, coronary luminal changes can be observed successibly for considerable length during single observation (Fig.1).

### Subjects

From April, 1997 to December, 1998, 32 patients were enrolled for coronary angioscopy. The patients were randomely divided into control and bezabibrate groups.

Oral administration of bezafibrate (Bezatol SR Kissei Co, 400 mg/day) was started immediately after the interventions and was continued for 6 months. The plaques in one or two epicardial arteries not targetted for interventions were observed during interventions. Six months later, angioscopy was repeated for observation of the identical plaques. Because of experimental nature, informed consent was obtained from all patients.

### Vulnerability Scores

Coronary atherosclerotic plaques are classified in color, into white, light yellow, white and non-glistening yellow on mosaic, non-glistening yellow (rarely brown) and glistening yellow [11,12,13]. Also, they are classified into regular (non-disrupted) and complex (disrupted) plaques [13,14]. Our angioscopic and pathologic studies revealed that glistening yellow plaques are vulneable and yellow one the next. Our prospective angioscopic follow-up study also confirmed that glistening yellow plaques are vulnerable [10].

Therefore, the vulnerability score of plaques was determined based on their color and surface morphology and it was compared before and 6 months later (Table I).

### Angiographic Measurement

Percentage stenoses were calibrated using cineangiograms and were compared before and 6 months later. An increase of 25% and a decrease of 25% in % stenoses were determined as progression and regression, respectively, and they were compared between the control and bezafibrate groups.

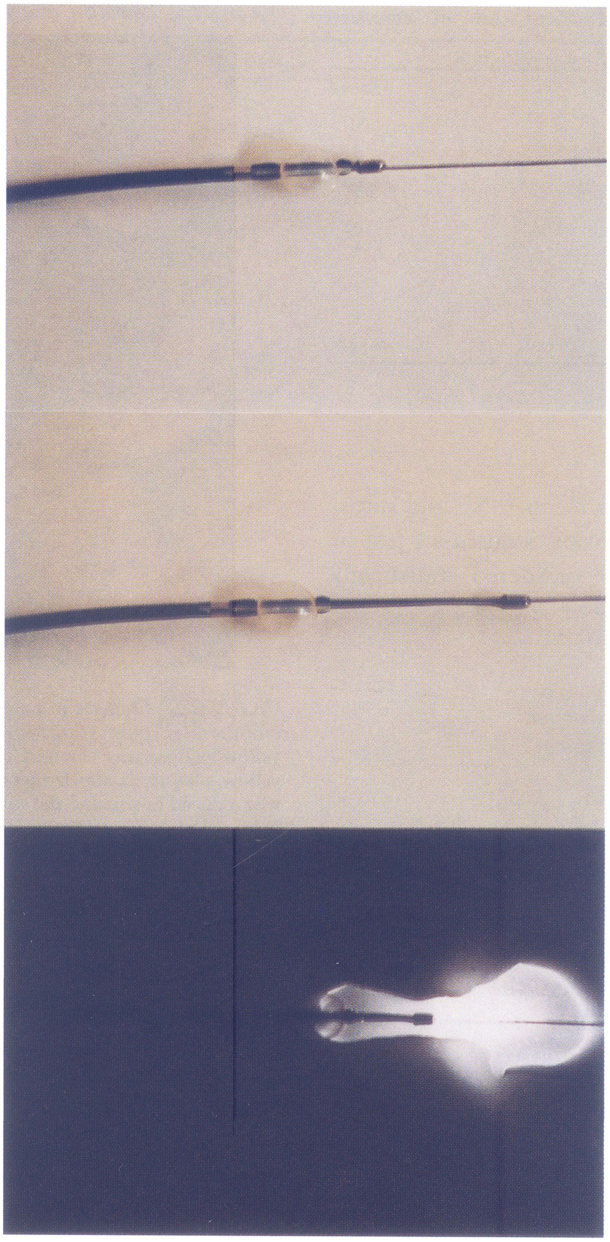

FIGURE 1 Angioscope used in this study. Top: fiberscope pulled back. Middle: fiberscope advanced. Bottom: during saline flush.

**Plasma Lipids Measurements**

Plasma total cholesterol (T.Chol), triglyceride (TG) and high density lipoprotein (HDL) were measured before initial and repeated angioscopy and the relationships between angioscopic changes and those in plasma lipids were examined.

TABLE I Angioscopic vulnerability score of coronary plaques

| *A. Plaque color* | Score |
|---|---|
| white | 0 |
| light yellow | 1 |
| white and yellow speckled | 1 |
| yellow | 2 |
| glistening yellow | 3 |
| *B. Surface morphology* | |
| regular | 0 |
| complex | 1 |

### Statistical Analysis

All datas were expressed as mean ± SE and statistical analyses were made using Student's t test or $x^2$ test and $p < 0.05$ was considered statistically significant.

### RESULTS

### Angioscopic Changes

Of 32 patients, 14 plaques in 10 control patients and 21 plaques in 14 patients were successfully observed during and 6 months after interventions. No significant differences were observed in patient's background between the control and bezafibrate groups (Table II).

Figure 2 shows representative examples of the changes in coronary plaques in the treated group. In Fig. 2A, a glistening yellow complex (rough surfaced) plaque before was changed into non-glistening yellow regular plaque (vulnerability score from 4 to 2). In Fig. 2B, a non-glistening yellow regular plaque disappeared almost completely and the luminal surface became white (vulnerability score from 2 to 0). In Fig. 2C, a yellow and brown colored plaque was reduced in size and the neighbouring surface changed from light yellow into white (vulnerability score from 2 to 1). The carina at the bifurcation was somewhat thickened, indicating fibrotic thickening. Thus, vulnerability score was reduced (from 1.6 to 0.8; $p < 0.05$) in bezafibrate group and was unchanged (from 1.4 to 1.3; NS) in control group (Fig. 3).

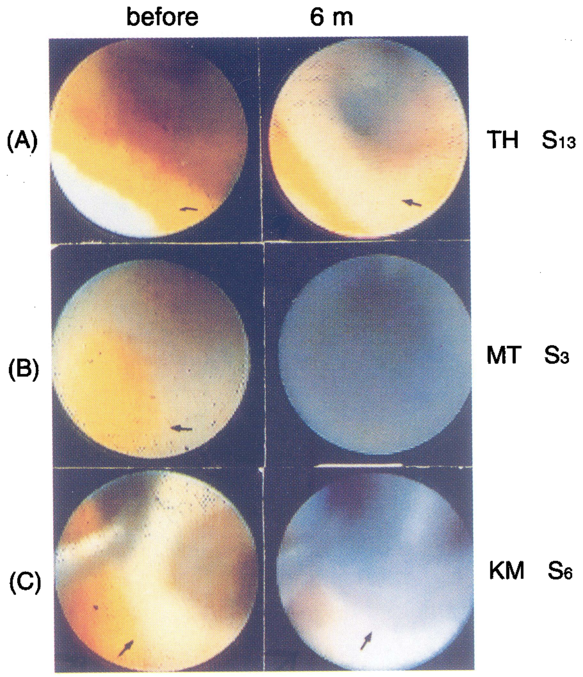

FIGURE 2 Changes in coronary plaques before (left) and 6 months after (right) treatment with bezafibrate. (A) a glistening yellow plaque was changed into yellow. (B) a non-glistening yellow plaque disappeared (absorbed?). (C) a yellow plaque was reduced in size and the surrounding portions changed from light yellow into white in color.

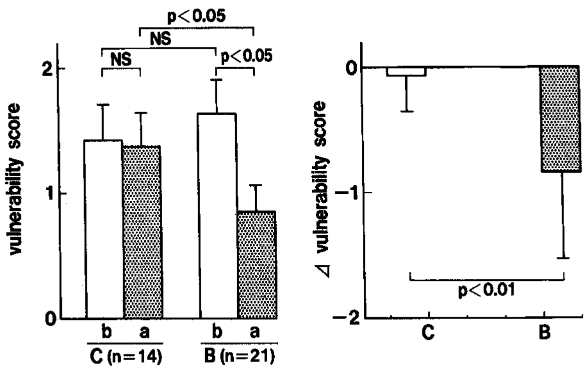

FIGURE 3 Changes in vulneability score. b: before. a: 6 months after. C: control group. B: bezafibrate group. NS: not significant.

TABLE II Background

| Characteristics | Control group (n = 10) | Bezafibrate group (n = 14) | *p* value |
|---|---|---|---|
| Age (y.o; means ± SE) | 63.5 ± 2.8 | 61.2 ± 2.5 | NS |
| Gender (F/M) | 0/10 | 2/12 | NS |
| Angina pectoris | 4 | 9 | NS |
| Acute myocardial infarction | 5 | 2 | NS |
| Old myocardial infarction | 1 | 3 | NS |
| *Coronary risk factors* | | | |
| hypertension | 2 | 2 | NS |
| current smoker | 3 | 4 | NS |
| hyperlipidemia | 3 | 8 | NS |
| diabetes mellitus | 2 | 4 | NS |
| *Medication* | | | |
| antiplatelet drugs (aspirin) | 9 | 14 | NS |
| long acting nitrate | 2 | 4 | NS |
| beta-blockers | 1 | 3 | NS |
| calcium antagonists | 7 | 10 | NS |
| ACE inhibitors | 1 | 2 | NS |
| potassium channel openor | 6 | 5 | NS |
| SBP (mmHg) | 143.6 ± 5.9 | 138.6 ± 5.3 | NS |
| DBP (mmHg) | 82.6 ± 3.7 | 83.4 ± 2.4 | NS |
| HR (bpm) | 75.4 ± 2.9 | 71.9 ± 1.8 | NS |
| *Plasma lipids* | | | |
| T Chol (mg/dl) | 206.5 ± 8.7 | 222.0 ± 10.8 | NS |
| TG (mg/dl) | 140.8 ± 14.4 | 180.2 ± 21.0 | NS |
| HDL (mg/dl) | 41.5 ± 2.8 | 40.9 ± 2.6 | NS |
| *Vessel disease* | | | |
| 0 | 1 | 2 | NS |
| 1 | 6 | 7 | NS |
| 2 | 2 | 4 | NS |
| 3 | 1 | 1 | NS |
| *Coronary intervention* | | | |
| not performed | 2 (2) | 3 (3)* | NS |
| POBA | 2 (1) | 8 (7) | NS |
| STENT | 7 (7) | 4 (4) | NS |

*: number of intervention sites (number of patients),
NS: not significant.

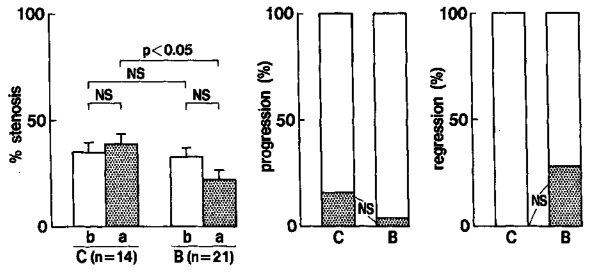

FIGURE 4 Angiographic changes.

In bezafibrate group, % stenosis assessed by angiography was decreased. However, no significant differences were observed in progression or regression assessed by angiography between the two groups (Fig. 4).

Two or more plaques with vulnerability score of 1 or more were observed in 4 patients treated. In 2 of these patients, vulnerability score was decreased in one plaque but was unchanged in others (Fig. 5).

| Color of plaque | patient | A | B | C | D | E | F |
|---|---|---|---|---|---|---|---|
| | white | | | | | ○○ | ○ |
| | light yellow or speckled | ● | ● | ● ○ | | ○ | |
| | yellow | ● | ● | ● | ● ○ | | ● |
| | glistening yellow | | | | | | |

FIGURE 5 Differential changes in color of regular plaques (vulnerability score) in the same patients. Open circle: unchanged. Solid circle: reduced in score.

### Changes in Plasma Lipids

The value of T Chol was unchanged and that of TG was decreased. On the contrary, the value of HDL was increased in bezafibrate group but were unchanged in control group (Fig. 6). In bezafibrate

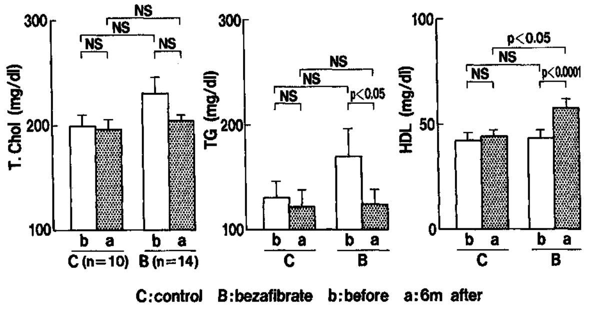

FIGURE 6 Changes in plasma lipids in control and bezafibrate group.

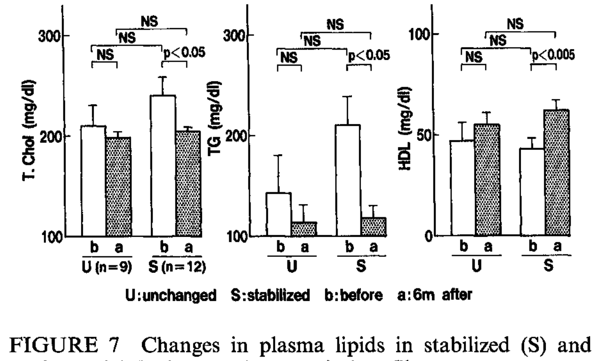

FIGURE 7 Changes in plasma lipids in stabilized (S) and unchanged (U) plaque subgroups in bezafibrate group.

group, both T Chol and TG were decreased and HDL was increased in patients with reduced vulnerability score but were unchanged in those with unchanged score (Fig. 7).

### DISCUSSION

The results in this study indicate that 6 month's oral administration of bezafibrate reduced vulnerability score. In our pathologic study, glistening yellow plaques had very thin cap covering the lipid pool. The cap was almost devoid of normal collagen fibers which act against disruption. Furthermore, small calcium crystals and ceroids, intermediate degradets of lipid produced by macrophages, as well as lipids, were deposited in the cap. Ex vivo angioscopic studies revealed that glistening is due to reflection of illumination at least by small calcium cristals, and existence of such hard substance in collagen fiber deficient superficial layer may be easily damaged by intraluminal pressure or flow changes, spasm, or distortion by cardiac contraction, resulting in disruption.

Yellow plaques were classified into lipid pool and non-lipid pool types. The changes common to both were superficial depositions of lipid and abnormal or deficient collagen fibers. Therefore, we considered that this category of plaques are more vulnerable than non-atherosclerotic or fibrotic plaques which angioscopically exhibit milky or white color [11,12].

In this study, a glistening yellow plaque was changed into non-glistening yellow plaques after 6 month's administration of bezafibrate. Although fibrotic responses to the mechanical damages induced by guide wire passing over the plaque during observation can not be denied, it is likely that the agent reduced lipid at least in the cap and the cap became thick due to increased collagen fibers or extracellar matrix. Non-glistening yellow plaques were also changed into light yellow or white, namely vulnerability score was reduced in the treated group. All these facts suggest that vulnerable plaques at least in the segments with angiographically insignificant stenoses can be stabilized or regressed by oral administration of bezafibrate. Because of long term follow-up study, observation was limited to the plaques in the segments without significant stenoses. Therefore, it remains to be elucidated whether those in significantly stenotic segments are changed similarly.

Although % stenosis assessed by angiography was also reduced in the treated group. However, angiographically determined regression or progression was not correlated with angioscopic changes. Therefore, it is beyond angiography for assessement of plaque stabilization.

Two or more plaques with vulnerability score of 1 or more could repeatedly be observed in same 4 patients. In 2 of them, one plaque was stabilized but not in other, suggesting different susceptibility of plaques to bezafibrate. The reason for this remains to be elucidated.

In bezafibrate group, TG was reduced and HDL was increased significantly but T Chol was unchanged. The treated group was divided into stabilized (at least one plaque was stabilized) and unstabilized groups and the changes in plasma lipids were compared. It was revealed that T Chol and TG were reduced and HDL was increased in the former group and none of them were changed in non-stabilized group, suggesting that significant improvement in plasma lipids is at least necessary for plaque stabilization. However, in individual patients, improvement in plasma lipids did not necessarily mean plaque stabilization, indicating participation of other unknown factors in stabilization.

Since angioscopy is an interventional and rather subjective diagnostic tool, other non-interventional and more objective tools, including measurement of plasma factors which selectively reflect vulnerability and also non-interventional therapeutic means should be developed in future to minimize the great burden of human beings, acute coronary syndromes.

### *References*

[1] Waters, D., Higgins, L., Gladstone, P., Kimball, B., Lamay, M. and Lesperance, J. Effect of lovastatin upon the evolution of coronary atherosclerosis as assessed by serial quantitative coronary arterigraphy. *J. Am. Coll. Cardiol.* 1993; **21**: 1312–1318.

[2] The Scandinavian Simvastatin Survival Study Group: Randomised trial of cholesterol lowering in 4444 patients with coronary heart disease: the Scandinavian Simvastatin Survival Study (4S). *Lancet.* **344**: 138.

[3] Shepherd, J., Cobbe, S.M., Ford, I., Isles, C.G., Lorimer, A.R., Macfarlene, P.W., McKillop, J.H. and Packerd, C.J. Prevention of coronary heart disease with pravastatin in men with hypercholesterolemia. *New Engl. J. Med.* 1995; **333**: 1301–1307.

[4] Jakema, J.W., Bruschke, A.V.G., van Boven, A.J., Reiber, J.H.C. and Bai, E.T. REGRESS Study Group: Effects of lipid lowering by pravastatin on progression and regression of coronary artery disease in symptomatic men with normal to moderately elevated serum cholesterol levels. *Circulation* 1995; **91**: 2528–2540.

[5] Ericsson, C.G., Hamsten, A., Nilsson, J., Grip, L., Svane, B. and deFaire, U. Angiographic assessment of effects of bezafibrate on progression of coronary artery disease in young male postinfarction patients. *Lancet* 1996; **347**: 849–853.

[6] Ericsson, C.G., Nilsson, J., Grip, L., Svane, B. and Hamsten, A. Effects of bezafibrate treatment on five years on coronary plaques causing 20% to 50% diameter narrowings (The Bezafibrate Atherosclerosis Intervention Trial). *Am. J. Cardiol.* 1997; **80**: 1125–1129.

[7] Ericsson, C.G. Results of the bezafibrate coronary atherosclerosis trial (BECAIT) and an update on trials now in progress. *Europ. Heart J.* 1998; **19**: H37–41.

[8] Yutani, C. and Hao, H. Histopathological study of acute myocardial infarction and pathoetiology of coronry thrombosis: A comparative study in four districts in Japan. *Jpn. Circ. J.* 1987; **51**: 352.

[9] Hao, H. and Yutani, N. Histopathological aspects of coronary thrombosis and its progression. *In Coronary Clinic* 1994; pp. 121–127.

[10] Uchida, Y., Nakamura, F., Tomaru, T., Morita, T., Oshima, T., Sasaki, T., Morizuki, S. and Hirose, J. Prediction of acute coronary syndromes by percutaneous coronary angioscopy in patients with stable angina. *Am. Heart J.* 1995; **130**: 204–211.

[11] Uchida, Y., Kanai, M., Uchida, H., Kameda, N. and Hiruta, K. Angioscopic classification of coronary plaques and its pathological correlations. *Coronary* 1999; **16**: 302–313.

[12] Uchida, Y., Takeuchi, K., Kanai, M., Sakurai, T., Kameda, N. , Hiruta, K., Tachihara, K. and Nagaki, K. Glistening yellow plaque, a vulnerable plaque, and its pathological correlations. *Circulation* 1999; **100**(Suppl 1): 514.

[13] Uchida, Y. Angioscopic detection of vulnerable plaques and prediction of acute coronary syndromes. In the Vulnerable Atherosclerotic Plaque, *Futura. Publish. Co.* 1999; NY.

[14] Sherman, C.T., Litback, F. and Grundfest, W. Coronary angioscopy in patients with unstable angina pectoris. *New Engl. J. Med.* 1986; **315**: 913–919.

